# Transitional Care Support for Medicaid-Insured Patients With Serious Mental Illness: Protocol for a Type I Hybrid Effectiveness-Implementation Stepped-Wedge Cluster Randomized Controlled Trial

**DOI:** 10.2196/64575

**Published:** 2024-11-12

**Authors:** J Margo Brooks Carthon, Heather Brom, Kelvin Eyram Amenyedor, Michael O Harhay, Marsha Grantham-Murillo, Jacqueline Nikpour, Karen B Lasater, Daniela Golinelli, Pamela Z Cacchione, Amanda P Bettencourt

**Affiliations:** 1 Center for Health Outcomes and Policy Research School of Nursing University of Pennsylvania Philadelphia, PA United States; 2 Leonard Davis Institute of Health Economics University of Pennsylvania Philadelphia, PA United States; 3 Institute for Medical Informatics and Biostatistics Perelman School of Medicine University of Pennsylvania Philadelphia, PA United States; 4 Penn Medicine at Home University of Pennsylvania Philadelphia, PA United States; 5 Nell Hodgson Woodruff School of Nursing Emory University Atlanta, GA United States; 6 New Courtland Center for Transitions and Health School of Nursing University of Pennsylvania Philadelphia, PA United States; 7 Penn Presbyterian Medical Center Philadelphia, PA United States; 8 Penn Implementation Science Center University of Pennsylvania Philadelphia, PA United States

**Keywords:** serious mental illness, health care disparities, Medicaid, evidence-based practice, implementation science, socioeconomic disparities in health

## Abstract

**Background:**

People diagnosed with a co-occurring serious mental illness (SMI; ie, major depressive disorder, bipolar disorder, or schizophrenia) but hospitalized for a nonpsychiatric condition experience higher rates of readmissions and other adverse outcomes, in part due to poorly coordinated care transitions. Current hospital-to-home transitional care programs lack a focus on the integrated social, medical, and mental health needs of these patients. The Thrive clinical pathway provides transitional care support for patients insured by Medicaid with multiple chronic conditions by focusing on posthospitalization medical concerns and the social determinants of health. This study seeks to evaluate an adapted version of Thrive that also meets the needs of patients with co-occurring SMI discharged from a nonpsychiatric hospitalization.

**Objective:**

This study aimed to (1) engage staff and community advisors in participatory implementation processes to adapt the Thrive clinical pathway for all Medicaid-insured patients, including those with SMI; (2) examine utilization outcomes (ie, Thrive referral, readmission, emergency department [ED], primary, and specialty care visits) for Medicaid-insured individuals with and without SMI who receive Thrive compared with usual care; and (3) evaluate the acceptability, appropriateness, feasibility, and cost-benefit of an adapted Thrive clinical pathway that is tailored for Medicaid-insured patients with co-occurring SMI.

**Methods:**

This study will use a prospective, type I hybrid effectiveness-implementation, stepped-wedge, cluster randomized controlled trial design. We will randomize the initiation of Thrive referrals at the unit level. Data collection will occur over 24 months. Inclusion criteria for Thrive referral include individuals who (1) are Medicaid insured, dually enrolled in Medicaid and Medicare, or Medicaid eligible; (2) reside in Philadelphia; (3) are admitted for a medical diagnosis for over 24 hours at the study hospital; (4) are planned for discharge to home; (5) agree to receive home care services; and (6) are aged ≥18 years. Primary analyses will use a mixed-effects negative binomial regression model to evaluate readmission and ED utilization, comparing those with and without SMI who receive Thrive to those with and without SMI who receive usual care. Using a convergent parallel mixed methods design, analyses will be conducted simultaneously for the survey and interview data of patients, clinicians, and health care system leaders. The cost of Thrive will be calculated from budget monitoring data for the research budget, the cost of staff time, and average Medicaid facility fee payments.

**Results:**

This research project was funded in October 2023. Data collection will occur from April 2024 through December 2025. Results are anticipated to be published in 2025-2027.

**Conclusions:**

We anticipate that patients with and without co-occurring SMI will benefit from the adapted Thrive clinical pathway. We also anticipate the adapted version of Thrive to be deemed feasible, acceptable, and appropriate by patients, clinicians, and health system leaders.

**Trial Registration:**

ClinicalTrials.gov NCT06203509; https://clinicaltrials.gov/ct2/show/NCT06203509

**International Registered Report Identifier (IRRID):**

DERR1-10.2196/64575

## Introduction

### Background

Approximately 10 million adults in the United States have a serious mental illness (SMI; ie, major depressive disorder, schizophrenia, and bipolar disorder) [1]. Medicaid, as the largest payer of mental health services, covers 26% of nonelderly adults with SMI [2]. However, up to half of Medicaid-insured individuals with comorbid SMI experience unmet treatment needs [3]. Our population of interest is Medicaid-insured individuals who are hospitalized for a medical condition but live with comorbid SMI. They experience worse health outcomes, particularly following hospitalization due in part to care fragmentation and a lack of connection to community-based services [4,5]. Such disparities are rooted not only in the complexities of their psychiatric conditions but also in a health care system that often fails to provide comprehensive and integrated care. Hospital-to-home transitional care programs can improve postdischarge outcomes through a focus on continuity of care; however, most of these programs often focus on specific medical conditions and lack comprehensive plans to address co-occurring mental health diagnoses [6-8]. Similarly, SMI-focused transitional care programs often exhibit a critical gap; they do not also have a comprehensive plan to address co-occurring chronic medical conditions [9-11]. This oversight is significant given the complex needs of individuals with SMI, who frequently face concurrent medical conditions that require integrated care approaches. In addition, existing transitional care models for patients with SMI who are admitted for psychiatric or nonpsychiatric reasons do not adequately address the social determinants of health (SDOH), a critical driver of optimal health and well-being [12,13]. The SDOH, including factors such as housing stability, employment, social support, and access to nutritious food, significantly influences health outcomes yet remains peripheral in many transitional care strategies. The absence of a holistic approach in these transitional care models underscores the necessity for integrating SMI care with physical health management and addressing the SDOH.

**Figure 1:**
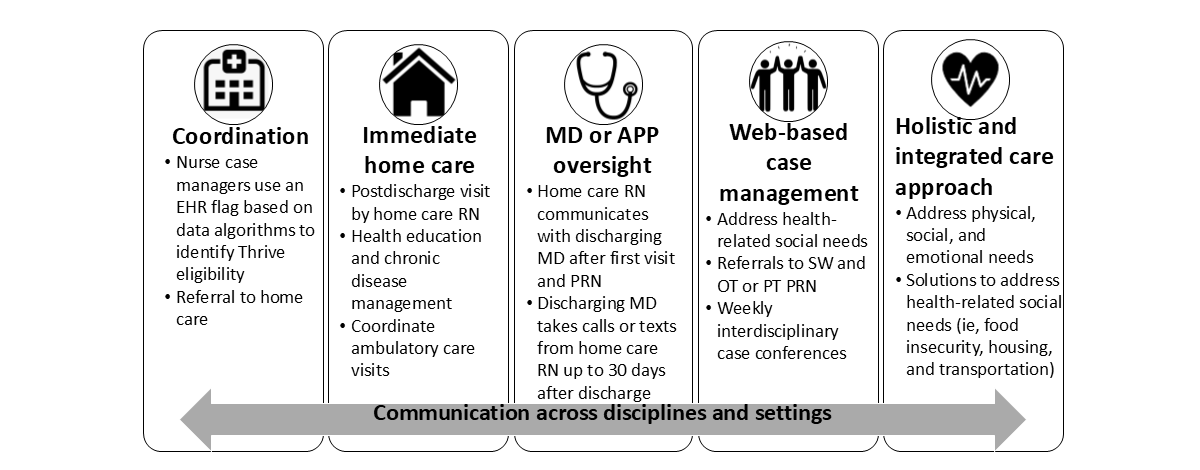
Components of the Thrive clinical pathway. APP: advanced practice provider; EHR: electronic health record; MD: medical doctor; OT: occupational therapy; PRN: pro re nata (as needed); PT: physical therapy; RN: registered nurse; SW: social worker.

The Thrive clinical pathway addresses the SDOH for Medicaid-insured individuals with multiple chronic conditions transitioning from hospital to home through intensive virtual case coordination, home care services, connections to community-based services, and supervision provided by the discharging clinician for up to 30 days (Figure 1).

Through this process, Thrive increases connections to community-based services and providers (ie, primary and specialty care) and decreases unfavorable posthospital use (ie, readmissions and emergency department [ED] visits). In a pilot study comparing 42 Thrive participants to 437 patients receiving usual care, we found a 50% reduction in 30-day hospital readmissions and a 50% increase in access to postdischarge primary care and specialty care [14].

### Enhancing Thrive: Integrating Serious Mental Illness

During the successful launch of Thrive at a single site, we also noted a sizable co-occurrence of SMI among our Thrive participants. This raised concerns over additional barriers they might face following hospitalization [15] and whether they experienced similar benefits from the Thrive intervention compared with participants without an SMI diagnosis. In an observational pilot study comparing utilization outcomes of Thrive participants with and without SMI, there were no statistically significant differences between the groups for 30-day readmissions and ED use. However, in qualitative interviews, Thrive participants revealed that while they were happy with the care provided for their medical conditions and with connections to social services, their behavioral health needs were not fully assessed or addressed (JM Brooks Carthon, 2024, unpublished data).

Building on these pilot findings, we now seek to adapt Thrive to ensure suitability for all patients insured by Medicaid including those with co-occurring SMI. Therefore, we are conducting a type I hybrid effectiveness-implementation, stepped-wedge, cluster randomized controlled trial at a new clinical site [16]. Our study is informed by Woodward’s Health Equity Implementation Framework (HEIF), which centers equity within the implementation process and focuses on the culturally relevant factors of recipients, the patient-provider interaction, and the societal and organizational context [17].

### Aims and Hypotheses

The specific aims of this research are to engage staff and community advisors in participatory processes to (1) prioritize additional components to the current Thrive care model suitable for participants with co-occurring SMI and evaluate the context required for adaptation of the intervention; (2) examine utilization outcomes (ie, Thrive referral; readmission; and ED, primary, and specialty care visits) for Medicaid-insured individuals with and without SMI who receive Thrive compared with usual care—we hypothesize that individuals with SMI who receive usual care will have worse utilization outcomes compared with those without SMI and that all individuals receiving Thrive with and without SMI will have better outcomes compared with those with usual care; and (3) evaluate the acceptability, appropriateness, feasibility, and cost-benefit of an adapted Thrive clinical pathway that is tailored for Medicaid-insured patients with co-occurring SMI.

## Methods

### Overview

This study will use a prospective, type I hybrid effectiveness-implementation, stepped-wedge, cluster randomized controlled trial design. The advantage of this trial design is that it offers an efficient and practical way to introduce Thrive at a new clinical site while reducing the ethical concerns of withholding an intervention that has shown benefit for economically disadvantaged populations [14]. We will conduct our study in accordance with the Standard Protocol Items: Recommendations for Interventional Trials (SPIRIT) statement and the Consolidated Standards of Reporting Trials (CONSORT) extension for stepped-wedge cluster randomized trials [18,19]. These guidelines ensure rigorous and transparent reporting of our trial design and outcomes and the checklists are included as Multimedia Appendices 1 and 2. Our use of a mixed methods (quantitative + qualitative) study design prioritizes quantitative data to evaluate program referrals, outcomes, and fidelity. Qualitative data will enrich our understanding of barriers and facilitators faced by health care providers in implementing the expanded Thrive intervention, particularly with the addition of mental health services [20]. Taken together, the findings from both the quantitative and qualitative approaches will allow for future intervention refinement if needed and determining the implementation strategies associated with adoption and sustainability. Multimedia Appendix 3 provides a study overview, and the methodology of each aim will be described.

### Study Setting

This study is set at the Cedar location of the Hospital of the University of Pennsylvania (HUP), part of the University of Pennsylvania Health System and a new site to implement Thrive. HUP-Cedar is located in West Philadelphia and serves as a safety net primarily treating the most socially at-risk residents of Philadelphia. Over half the patients at this 45-bed hospital are Medicaid or dually Medicare and Medicaid insured, and the majority are from diverse racial and ethnic minority backgrounds.

### Aim-1 Methods

Aim 1 is to engage staff and community advisors in participatory processes to (1) prioritize additional components to the current Thrive care model suitable for participants with co-occurring SMI and (2) evaluate the context required for adaptation of the intervention. The preimplementation process used to meet aim 1 is described extensively elsewhere (JM Brooks Carthon, 2024, unpublished data).

### Aim-2 Methods

Aim 2 is to examine utilization outcomes for Medicaid-insured individuals diagnosed with and without SMI who receive Thrive services compared with usual care.

### Design and Randomization

The stepped-wedge cluster design accounts for the proportion of the study period that each group spends in the control and intervention periods (Table 1).

Randomization will occur at the unit level. There are 2 units at HUP-Cedar; using a random number generator, the first unit will be chosen. Eight weeks later, the second unit will begin Thrive referrals. Each unit has 1 case manager and 1 social worker responsible for discharge planning and initiating Thrive referrals. Due to the small number of clinicians and their shared workspace, all 4 will be trained simultaneously, and concealing allocation will not be feasible.

**Table 1:** Study diagram for the type I hybrid effectiveness-implementation, stepped-wedge, cluster randomized controlled trial of the adapted Thrive clinical pathway for serious mental illness.

Cluster	Baseline (3 months)	Enrollment period 1 (two months)	Enrollment period 2 (two months)	Follow-up (17 months)
Sequence 1 (first unit)		✓	✓	✓
Sequence 2 (second unit)			✓	✓

### Study Population

Eligibility criteria for Thrive referral include individuals who (1) are Medicaid insured, dually enrolled in Medicaid and Medicare, or Medicaid eligible; (2) reside in Philadelphia; (3) are admitted for a medical diagnosis for over 24 hours at the study hospital; (4) are planned for discharge to home; (5) agree to receive home care services; and (6) are aged 18 years or older. Patients with SMI will be identified using the *International Classification of Diseases, Tenth Revision* (*ICD-10*) codes for major depressive disorder, bipolar disorder, or schizophrenia in secondary diagnoses [21]. Exclusion criteria include referral to palliative or hospice home care services at discharge.

### Current Usual Care

Usual care includes a discharge to the patient’s home with standard discharge care planning that addresses the patient’s medical needs. This may or may not encompass referrals to home care and other outpatient services as appropriate.

### Thrive Intervention Group

Patients are referred to Thrive by nurse case managers or social workers during the hospital discharge planning process. They are alerted to a potential Thrive referral by an electronic health record (EHR) flag developed by our team [22]. Thrive participants agree to home care services through Penn Medicine at Home. A home care nurse visits the patient within 72 hours of discharge. This visit includes medication reconciliation, review of discharge orders, setting person-centered goals, and referral to other home care services (eg, physical therapy) as deemed necessary. The nurse also determines the visit frequency based on the patient’s needs, ranging from 1-3 visits per week. All Thrive participants receive a social work referral, and their psychosocial and mental health needs are further assessed and connections to community resources are provided. Home care nurses provide patient updates to the discharging provider (eg, a medical doctor or advanced practice provider) following the first home visit. Extended supervision by the discharging provider is provided for up to 30 days or until Thrive participants have been seen by their primary care provider. A key feature of Thrive is the intensive, equity-focused, 30-day case management provided during weekly virtual interdisciplinary case conferences. These meetings focus on assessing and addressing the SDOH such as housing, food insecurity, and substance use services as needed. If a source of primary care is absent, we work to link Thrive participants to community providers. Additional Thrive services will be provided as determined by the work group (Aim 1).

### Data Collection and Sources

**Figure 2:**
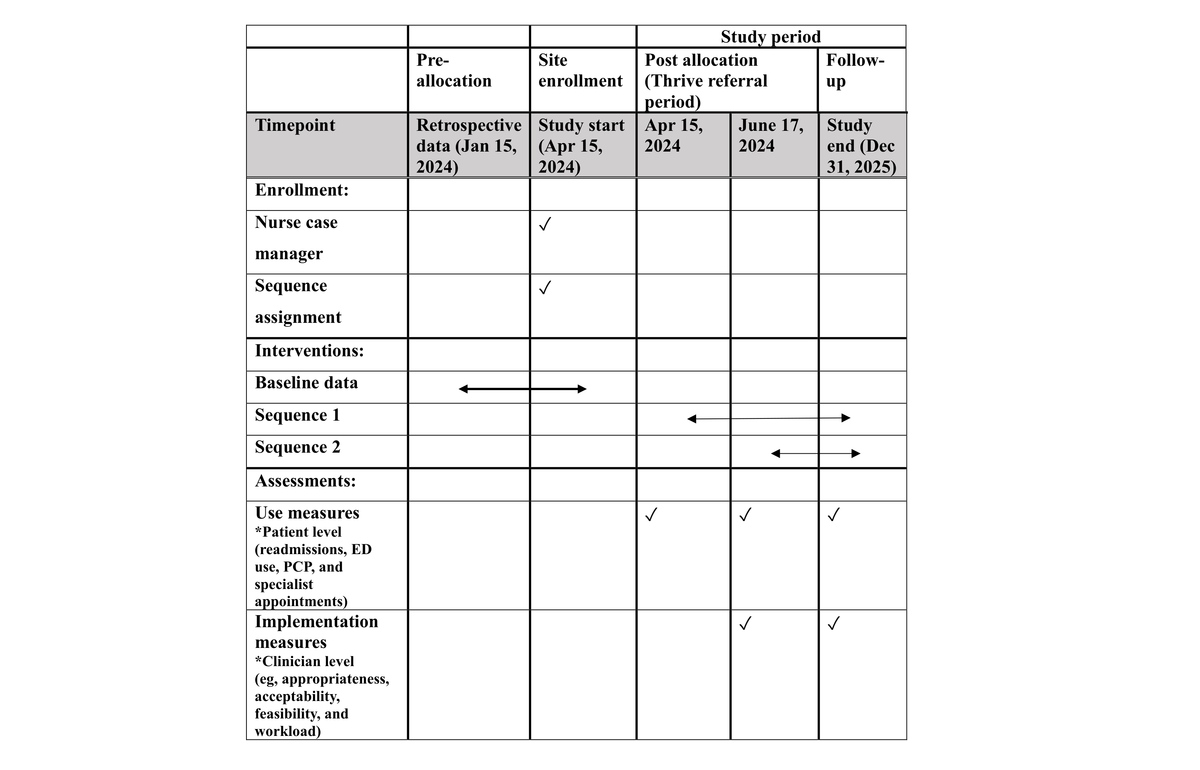
SPIRIT (Standard Protocol Items: Recommendations for Interventional Trials) diagram—Thrive schedule of evaluations. ED: emergency department; PCP: primary care provider.

Clinical data, outcome metrics, and postacute care service use will be derived from the EHR at baseline and throughout the Thrive referral period. The characteristics of Thrive participants will be captured in weekly case conferences and stored in a University of Pennsylvania REDCap (Research Electronic Data Capture; Vanderbilt University) system database. Data for the usual care group will be derived from the EHR. The study timeline is outlined in the SPIRIT diagram (Figure 2).

### Variables and Measures

The primary independent variable is participation in Thrive. The outcome variables include Thrive referral rates; 30-, 60-, and 90-day readmissions and ED visits; and receipt of primary and specialty care within 30 days of index hospitalization [23-25]. For Thrive participants, these outcomes will be assessed from the date of hospitalization that resulted in a referral to Thrive. For the usual care group, we will identify all readmission and ED visits occurring after the first index hospitalization identified in the EHR from the calendar year of the study. Additional covariate data at both the patient (eg, demographics, comorbidities, or mental health diagnoses) and unit levels (eg, type of unit, discharging team, or provider) will be collected.

### Analysis

Monthly reports will be generated to monitor referral patterns to the Thrive clinical pathway. The primary analysis will use a mixed-effects negative binomial regression model, accounting for both medical team and time effects, to evaluate readmission and ED utilization patterns comparing those with and without SMI who receive Thrive to those with and without SMI who receive usual care [26,27]. Sensitivity analyses may include logistic regression, depending on data distribution.

### Power Analysis

Based on a power analysis, a sample size of 472 (236 Thrive participants and 236 usual care) for the outcome of readmissions, or a sample of 534 (267 Thrive participants and 267 usual care) for the outcome of ED visits, will yield 80% power to detect significance at the *P*<.05 level. Our goal is a sample size of 534 to ensure adequate power for both outcomes. This assumes a 20% (106/534) loss to follow-up rate. Furthermore, assuming at least a third of the patients will have SMI and an overall 30-day readmission rate of 28% (44/160), a sample of 534 patients will have 80% power to detect an odds ratio as small as 1.46, comparing those with and without SMI, to be statistically significant at the *P*<.05 level. Power analyses were conducted using Power Analysis and Sample Size 2021 (NCSS Statistical Software) [28].

### Aim-3 Methods

Aim 3 is to evaluate the acceptability, appropriateness, feasibility, and cost-benefit of an adapted Thrive clinical pathway that is tailored for Medicaid-insured patients with co-occurring SMI.

#### Design

Guided by Woodwards’ HEIF and using a mixed methods approach, we will survey and interview patients, organizational leaders, and health care providers to determine if HEIF domains including cultural factors, clinical encounters, and societal context are associated with uneven or disparate benefits to the intervention.

#### Sample

##### Patients

Using a convenience sampling approach, the project manager (PM) will invite patients referred to Thrive to provide feedback on their experiences with the interventions. If Thrive participants agree, the PM will enroll and obtain consent shortly after discharge. Refusal to participate in an interview does not exclude the patient from getting Thrive services. We will attempt to recruit up to 40 patients for interviews (20 with SMI and 20 without). For participants with a history of psychosis, we will complete the Evaluation of the Capacity to Sign Consent to assess their ability to participate in the interviews or survey. Thrive participants will be excluded from interviews if they are (1) incapable of participating in a group discussion or understanding content as determined by the Evaluation of the Capacity to Sign Consent or (2) experiencing an acute cognitive impairment or intellectual disability.

##### Clinicians

Fifteen clinicians (eg, physicians, advanced practice providers, nurse case managers, and home care nurses) will be recruited to participate in interviews at the end of the study. These professionals will provide critical insights into the feasibility, appropriateness, and workload involved in referring patients to the expanded Thrive clinical pathway.

##### Health Care Leaders

We will recruit 5 leaders at HUP-Cedar and Penn Medicine at Home to assess the broader contextual factors affecting the acceptability and implementation of Thrive. These will take place at the end of the study.

#### Data Collection

##### Qualitative Data Collection

Interview guides will be developed using our previous experience piloting Thrive, the domains of the HEIF, and Proctor’s implementation outcomes. Questions will be tailored for patients and clinicians and may include perceptions of bias, culturally relevant care, discrimination, acceptability of Thrive, and feasibility of its delivery. During interviews, participants will be asked for permission to audio record the interview. If the participant consents to the interview but does not agree to recording, the interviewer will take detailed notes during the conversation. Demographics of clinicians and administrators will also be collected. Directly following the interview, the interviewer will record field notes to add details not apparent from a transcript. Audio recordings will be labeled with a unique ID number. They will be uploaded to a secure server and transcribed by ADA Transcription, a transcription company with considerable experience in qualitative research, HIPAA (Health Insurance Portability and Accountability Act) regulations, and confidentiality. Cleaned transcripts will be entered into NVivo 14 (Lumivero) for coding and analysis. All interview participants will receive a US $50.00 gift card for participation.

##### Quantitative Data Collection

At the beginning of their interviews, clinicians and patients will be asked to complete a 12-item survey based on Proctor’s implementation outcomes, assessing acceptability (how fair or reasonable Thrive is deemed), appropriateness (to what extent Thrive seems suitable), and feasibility (the practicality and ease of delivering Thrive). Each construct has a 4-item instrument with a 5-point Likert scale that asks participants to rate the degree of agreement or disagreement with each item. Each instrument is averaged with higher scores indicating greater acceptability, appropriateness, or feasibility [29].

##### Cost-Benefit Data

The cost of Thrive will be calculated from budget monitoring data for the research budget and the cost of staff time [30]. Postdischarge use (ie, readmissions and ED use) will be derived from the EHR. Average Medicaid facility fee payments per admission will be obtained from the Pennsylvania Health Care Cost Containment Council, an independent state agency responsible for addressing health costs [31].

#### Analysis

In our concurrent mixed methods approach, surveys and interviews will be collected at the same time. Summary statistics will be produced from the quantitative measures of acceptability, appropriateness, and feasibility. All interview data will be stored and analyzed on NVivo version 14 using constant comparative analysis to examine data across cases.

#### Triangulation of Quantitative and Qualitative Data

Using a convergent, parallel, mixed methods design [32-34], analyses will be conducted simultaneously for the surveys and interviews. This design will allow us not only to compare the quantitative and qualitative findings but also to validate and expand our quantitative results using qualitative data. Integration of the results will occur during analysis and interpretation to confirm or cross-validate findings. Using the HEIF as an organizing framework, we will look for linkages between our findings from the quantitative analysis and related themes that emerge from the qualitative data [35].

#### Cost-Benefit Analysis

The cost of Thrive will be calculated from budget monitoring data for the research budget and the cost of staff time [30]. We will use the number and acuity of inpatient admissions to estimate the costs paid by Medicaid for patients who received Thrive compared with usual care. In Pennsylvania, Medicaid pays by hospital admission; so, to estimate inpatient costs, we will use the average Medicaid facility fee payment per admission reported by the Pennsylvania Health Care Cost Containment Council and increase it by 17.7% to reflect typical Medicaid professional fees as a percentage of facility fees [36,37]. We will adjust this inpatient admission cost estimate to reflect the acuity of admissions as follows. First, we will estimate the acuity of each admission by multiplying its diagnostic-related group code by standardized case-mix weights published by the Centers for Medicare and Medicaid Services [31]. We will average these case-mix weights by study arm and divide them by the average case-mix weight for all Medicaid discharges in our dataset. We will multiply these arm-specific adjustment factors by the Medicaid cost per admission to arrive at an acuity-adjusted average cost of admission for each arm.

### Ethical Considerations

The study has received approval from the University of Pennsylvania institutional review board (#8, Protocol 854422 on October 4, 2023), including a HIPAA waiver of informed consent for clinical data derived from the EHR. All study data collected will be deidentified, and participants will be assigned a pseudoidentifier. No individual participant will be identifiable in any published materials. Data will only be accessible on password-protected computers available to the study team. Written consent will be obtained before patient, clinician, and health system leader interviews. Interview participants will be compensated US $50.00 by gift card. The study will be conducted according to national regulations and the Declaration of Helsinki**.**

### Dissemination

Our team will lead extensive efforts to disseminate our findings, emphasizing the expanded scope of Thrive to thoroughly address the mental health needs of patients with co-occurring SMI. We will use various channels, including publications; conference presentations; policy briefs; and engagement with both national and international networks through the Penn School of Nursing Communication Office, Leonard Davis Institute of Health Economics, and the Center for Health Outcomes and Policy Research. Planned dissemination activities include (1) up to 5 publications (at least 1-2 per aim); (2) annual scientific presentations and conferences; (3) infographics and guest appearances on relevant podcasts (eg, Amplify Nursing); and (4) development of a comprehensive Thrive website, featuring open-access modules on the components and implementation processes of the expanded program. We will also engage with our Thrive Community Advisory Board quarterly to ensure our dissemination efforts are equity-focused and resonate with health system and community stakeholders.

## Results

Our research was funded in October 2023. Data collection began in April 2024 and is expected to continue through December 2025. As of October 17, 2024, a total of 46 patients have been referred to Thrive. We will conduct data analysis early in 2026. We anticipate publishing results in 2025-2027.

## Discussion

### Expected Findings

We anticipate that patients with and without co-occurring SMI will benefit from Thrive enrollment and experience less unfavorable use (ie, readmissions and ED visits) and more favorable use (ie, primary care, specialty, and connections to community-based resources) than those receiving usual care. We also anticipate the adapted version of Thrive to be deemed feasible, acceptable, and appropriate by patients, clinicians, and health system leaders.

### Comparisons with Previous Work

Transitional care programs that focus on patients with SMI most often do not include a comprehensive plan to address integrated medical and psychiatric conditions [9-11]. In addition, there is a lack of focus on addressing SDOH, which are driver of both physical and mental health outcomes. For example, programs like the mobile health transitions of care program for adults with schizophrenia spectrum disorders aim to support patients with SMI in the critical period following hospital discharge through mobile health technologies [38]. Similarly, models such as the Depression and Coleman Care Transitions Intervention focus specifically on mental health conditions like depression, without integrating care for co-occurring physical health issues [9].

### Strengths and Limitations

The major strength of our study is the use of a stepped-wedge, cluster randomized controlled trial design. This allows all eligible patients at our study hospital the opportunity to be referred to Thrive during the study window. One limitation is the use of EHR data for our usual care group, as we can only capture use that occurs within our health system.

### Conclusion

This study is poised to advance the field of equity-focused, evidence-based interventions by testing the effectiveness of Thrive in improving care transitions for patients with Medicaid insurance and co-occurring SMI and examining Thrive’s implementation at a new site. The infusion of equity-focused principles in the design and evaluation of Thrive is anticipated to contribute substantially to both implementation and effectiveness outcomes, offering critical insights into how these principles can enhance health care delivery for patients with the highest health-related social needs.

## Appendix

Multimedia Appendix 1 Reporting checklist for protocol of a clinical trial based on the SPIRIT (Standard Protocol Items: Recommendations for Interventional Trials) guidelines.

Multimedia Appendix 2 CONSORT (Consolidated Standards of Reporting Trials) checklist for stepped wedge trials.

Multimedia Appendix 3 Summary of outcomes, measures, data sources, collection method, and time.

Multimedia Appendix 4 Peer Review Feedback from the Agency for Healthcare Research and Quality.

## Abbreviations

CONSORT: Consolidated Standards of Reporting Trials
ED: emergency department
EHR: electronic health record
HEIF: Health Equity Implementation Framework
HIPAA: Health Insurance Portability and Accountability Act
HUP: Hospital of the University of Pennsylvania
ICD-10: International Classification of Diseases, Tenth Revision
PM: project manager
SDOH: social determinants of health
SMI: serious mental illness
SPIRIT: Standard Protocol Items: Recommendations for Interventional Trials

## Data Availability

The datasets generated and analyzed during this study are not publicly available due to their inclusion of patient identifiers and would need to be requested through the Penn Data Store, as they are derived from the electronic health record (EHR).
