# Supplementary material for: Transitional Care Support for Medicaid-Insured Patients With Serious Mental Illness: Protocol for a Type I Hybrid Effectiveness-Implementation Stepped-Wedge Cluster Randomized Controlled Trial
Source: JMIR Res Protoc. 2024 Nov 12;13:e64575. doi: 10.2196/64575 (PMC11599882; doi:10.2196/64575)
Supplement: Multimedia Appendix 3 [file resprot_v13i1e64575_app3.docx]

**Summary of outcomes, measures, data sources, collection method, and time**

| Construct | Design | Measure | Method of Collection | Timing |
| --- | --- | --- | --- | --- |
| **Aim 1.** Engage staff and community advisors in participatory processes to: a). prioritize additional components to the current Thrive care model suitable for participants with co-occurring SMI and b). evaluate the context required for adaptation of the intervention. | | | | |
| Perceptions of the essential aspects of the adapted Thrive clinical pathway | The group will form using principles of Human Centered Design and prioritize the adaptation of Thrive | Perceptions of the feasibility of the intervention in the current setting before rollout, and their views on the value of the Workgroup | Semin-structured interviews of Workgroup members and other identified stakeholders | At the end of Thrive adaptation period |
| **Aim 2.** Examine utilization (i.e., referral, readmission, and ED) and connections to primary and specialty care for Medicaid-insured individuals with and without SMI who receive Thrive compared to usual care**.** | | | | |
| Thrive referrals | Stepped wedge cluster randomized controlled trial | Proportion of Thrive referrals of those who are Thrive eligible | Electronic health record (EHR) | Baseline and at study end |
| 30-, 60-, 90-day readmissions |  | Proportion of Thrive participants experiencing the outcome within 30-, 60-, 90-days of discharge compared to usual care group with and without SMI |  |  |
| 30-, 60-, 90-day ED visits |  |  |  |  |
| Primary care visit within 30 days of discharge |  | Proportion of Thrive participants experiencing the outcome within 30-days of discharge compared to usual care group with and without SMI |  |  |
| Specialty care visit within 30 days of discharge |  |  |  |  |
| **Aim 3.** Evaluate the acceptability, appropriateness, feasibility, and cost-benefit of an adapted Thrive clinical pathway that is tailored for Medicaid-insured patients with co-occurring SMI. | | | | |
| Culturally relevant factors | Mixed methods approach to determine if HEIF factors are associated with uneven or disparate benefits to the intervention and to assess the implementation of Thrive at a new site | Social concerns of Thrive participants and organization’s commitment to addressing them | Interviews with patients, clinicians, and administrators involved with Thrive | Study end |
| Clinical encounter |  | Relative advantage of Thrive to patients, degree of fit with existing practice, competing demands and bias |  |  |
| Societal context |  | Structures outside of the hospital that affect patient care |  |  |
| Acceptability |  | how fair or reasonable Thrive is deemed | Survey and interviews with clinicians and administrators | Study end |
| Appropriateness |  | To what extent Thrive seems suitable |  |  |
| Feasibility |  | The practicality and ease of delivering Thrive |  |  |
| Cost-benefit analysis |  | Cost of Thrive compared to cost of readmissions | EHR & state cost data | Study end |
