# Supplementary material for: Transitional Care Support for Medicaid-Insured Patients With Serious Mental Illness: Protocol for a Type I Hybrid Effectiveness-Implementation Stepped-Wedge Cluster Randomized Controlled Trial
Source: JMIR Res Protoc. 2024 Nov 12;13:e64575. doi: 10.2196/64575 (PMC11599882; doi:10.2196/64575)
Supplement: Multimedia Appendix 4 [file resprot_v13i1e64575_app4.pdf]

SUMMARY STATEMENT
(Privileged Communication)

PROGRAM CONTACT:
William Freeman
(301) 427-1320
william.freeman@ahrq.hhs.gov

Release Date: 08/02/2023
Revised Date:

Application Number: 1R18HS029815-01

Principal Investigator

BROOKS CARTHON, JACQUELINE MARGO

Applicant Organization: UNIVERSITY OF PENNSYLVANIA

Review Group: ZHS1 HSR-B (02)
AHRQ Special Emphasis Panel
Dissemination and Implementation of Equity-Focused Evidence-Based Interventions in Healthcare Delivery Systems

Meeting Date: 07/11/2023
Council: OCT 2023
Requested Start: 09/01/2023

Opportunity Number: RFA-HS-23-002
PCC: OEREP

Project Title: An equity-focused intervention to improve care transitions for Medicaid insured individuals with co-occurring serious mental health
SRG Action: Impact Score:55

Human Subjects: 30-Human subjects involved - Certified, no SRG concerns
Animal Subjects: 10-No live vertebrate animals involved for competing appl.
Gender: 1A-Both genders, scientifically acceptable
Minority: 1A-Minorities and non-minorities, scientifically acceptable
Clinical Research - not NIH-defined Phase III Trial

Project Year	Direct Costs Requested	Estimated Total Cost
1	302,678	489,629
2	312,294	505,185
3	312,294	505,185
TOTAL	927,266	1,499,999

ADMINISTRATIVE BUDGET NOTE: The budget shown is the requested budget and has not been adjusted to reflect any recommendations made by reviewers. If an award is planned, the costs will be calculated by Institute grants management staff based on the recommendations outlined below in the COMMITTEE BUDGET RECOMMENDATIONS section.

1R18HS029815-01 BROOKS CARTHON, JACQUELINE

RESUME AND SUMMARY OF DISCUSSION: This application, submitted by Jacqueline Brooks Carthon, PhD, from the University of Pennsylvania was in response to HS23-002 and was titled, "An equity-focused intervention to improve care transitions for Medicaid insured individuals with co-occurring serious mental health." The investigator proposes to adapt, implement, and evaluate the THRIVE post discharge support clinical pathway to determine if the invention benefits people insured with Medicaid with and without serious mental illness (SMI) equally and to evaluate the organizational features that support its successful implementation. The specific aims of the project are to (1) engage staff and community advisors in participatory implementation processes to consider components of the EOHC framework to integrate into THRIVE and evaluate the context required for adaptation of the THRIVE Clinical Pathway for all Medicaid-insured patients, including those with SMI, (2) Examine utilization outcomes (i.e., THRIVE referral, readmission, ED, primary and specialty care visits) for Medicaid-insured individuals with and without SMI who receive THRIVE compared to usual care, and (3) evaluate the acceptability, appropriateness, feasibility, and cost-benefit of an adapted THRIVE clinical pathway that is tailored for Medicaid-insured patients with co-occurring SMI. There were some strengths that were identified for this proposal. Individuals with SMI present to the hospital with poorly managed medical conditions compared with other patients and require more intensive transitional care coordination after a hospitalization. The PI Brooks-Carthon has expertise with respect to disparities and equity research and she was one of the developers of the THRIVE Clinical Pathway, the intervention studied in this proposal. The investigative team had complementary expertise with expertise in implementation science, cost evaluation, data management, statistical analysis, and equity-based design. Additionally, several investigators were involved in the original development of THRIVE. The plan to perform a cost-benefit analysis of THRIVE efforts showing rigor and practicality was viewed as a strength of the proposed approach. The environment was described as suitable for the proposed study and clear details were provided regarding the collaboration across institutions. There were several weaknesses that detracted from the panel's enthusiasm. The population of focus was unclear. The panel was uncertain whether the focus was all people insured by Medicaid or those insured by Medicaid and having an SMI. The proposal presents THRIVE to address unmet needs, but no description of these unmet needs is provided. The panel was concerned that no clear description of THRIVE was offered other than that it leverages technology with an EHR flag. There was concern that the team lacked SMI expertise, which was critical to the proposal. There was a concern that no one on the investigative team was employed by the implementing health system and/or no one from HUP-Cedar was on the investigative team. Given the embedded nature of the work, this could affect feasibility of implementation. The results of the pilot study were viewed as insignificant, and it was unclear how aim 2 in the proposed application differed from what had been achieved. These weaknesses reduced the panel's enthusiasm that the project will likely result in D&I evidence that can contribute to a better understanding of how to scale the intervention or apply it to other settings and populations. Overall, in consideration of the strengths and weaknesses discussed regarding the scientific and technical merit of this application, the reviewers gave this application a score of 55.

DESCRIPTION (provided by applicant): People insured by Medicaid with a co-occurring serious mental illness (i.e., depression, schizophrenia, and bipolar disorder) experience persistent and growing disparities following medical hospitalizations and interventions that focus on their transitional care needs are lacking. Our study focuses on the adaptation of the THRIVE, an equity-focused evidenced based intervention that supports Medicaid-insured individuals with multiple chronic conditions transitioning from hospital to home. THRIVE services include coordinating care, standardizing interdisciplinary communication, and addressing unmet clinical and social needs following hospital discharge. While evidence suggests robust improvements in post-discharge outcomes for THRIVE

participants, it remains unclear whether the interventions equally benefit patients with and without SMI. The goals of this project are to adapt, implement and evaluate THRIVE to ensure suitability for all patients insured by Medicaid including those with co-occurring SMI. To meet this goal, we propose the conduct of a Type I Hybrid effectiveness-implementation stepped wedge cluster randomized controlled trial (CRCT)15 at a new site, the Hospital of the University of Pennsylvania Cedar Campus (HUP-Cedar). HUP-Cedar is a 100-bed minority serving hospital located in West Philadelphia where >50% of patients are Medicaid or dually Medicare and Medicaid-insured. This study represents a three-way partnership among health services researchers, community advisors, and health care leadership and staff at HUP-Cedar and Penn Medicine at Home and seeks as its primary objective to leverage participatory methods to adapt the THRIVE intervention and evaluate the context required for implementation. A second objective for this study is to examine differences in the utilization outcomes (e.g., readmissions) between THRIVE participants with SMI compared to those receiving usual care. Finally, we will employ mixed methods approaches through participant and clinician interviews and surveys to evaluate the acceptability, appropriateness, feasibility and cost of the intervention. Our study and engagement with community and health system partners is informed equity centered frameworks that will help to emphasize the systems, policies and delivery process from which inequities might arise. If our study hypotheses are supported, we will identify not only if an equity-oriented evidence-based intervention can be adapted and implemented to meet the needs of Medicaid insured patients including those with SMI, but we will also uncover the factors associated with those results. Our proposal is well-aligned with AHRQ's goal to develop and adapt EFEBI's that prioritize the voices and needs of the most underserved populations and change care processes to accelerate equity within healthcare systems. Collectively, the results of this study will provide the foundation for the next phase of our research, which includes a multi-site evaluation of equity-based implementation strategies to scale and rigorously evaluate THRIVE diverse hospital settings.

PUBLIC HEALTH RELEVANCE: People insured by Medicaid with co-occurring serious mental illness (SMI) experience significant disparities following non-psychiatric hospitalizations. Our previously developed post discharge support pathway has successfully reduced post hospitalization disparities, though its benefits for patients diagnosed with SMI are unknow. This study seeks to adapt, implement, and evaluate the THRIVE post discharge support clinical pathway to determine if the invention benefits people insured with Medicaid with and without SMI equally and to evaluate the organizational features that support its successful implementation.

CRITIQUE 1

Significance:	5
Investigator(s):	4
Innovation:	3
Approach:	8
Environment:	2

CRITIQUE NOTE: The sections that follow are the essentially unedited, verbatim comments of the individual committee members assigned to review this application. The attached commentaries may not necessarily reflect the position of the reviewers at the close of group discussion, nor the final majority opinion of the group. The above RESUME/SUMMARY OF DISCUSSION represents the evaluation of the application by the entire committee.

Overall Impact: This R18 proposal will adapt, implement and evaluate THRIVE, an existing intervention developed by the PI and some team members, to ensure suitability for patients insured by Medicaid, including those with co-occurring SMI. Three Aims will be conducted:

Aim 1. Engage staff and community advisors in participatory implementation processes to consider components of the EOHC framework (equity-oriented health care) to integrate into THRIVE and evaluate the context required for adaptation of the THRIVE. The team proposes to form a working group comprised of our established Community Advisory Board, HUP-Cedar, and Penn Medicine home care clinicians to collaboratively adapt THRIVE to meet the needs of participants with SMI
Aim 2. Examine utilization outcomes (i.e., THRIVE referral, readmission, ED, primary and specialty care visits) for Medicaid-insured individuals with and without SMI who receive THRIVE compared to usual care. The team hypothesizes that individuals receiving THRIVE with and without SMI will have better outcomes compared to those with usual care. Aim 3. Evaluate the acceptability, appropriateness, feasibility, and cost-benefit of an adapted THRIVE. The team proposes to conduct surveys and interviews with patients, organizational leaders, and health care providers to elicit their experiences with the intervention and determine if domains including, cultural factors, clinical encounters, and societal context are associated with uneven or disparate benefits to the intervention. Tailoring an existing intervention is a promising approach. However, several weaknesses exist. The population of focus is unclear: SMI is a broad umbrella of diagnoses and conditions, which are very different and require different implementation approaches and adaptions for the THRIVE. The team plans to include patients with major depressive disorder, bipolar disorder, or schizophrenia, but this will lead to a very heterogeneous sample, requiring a different version of the adapted interventions. Because the team plans to exclude participants with cognitive impairments or those deemed with no capacity to consent, the study risks a serious bias of exclusion of individuals with schizophrenia and bipolar disorder. It is ambitious to adapt an intervention, including THRIVE, to several patient populations in one study. The suitability of the team to conduct the study and the fit of the environment (study site) and access to participants are unclear. The study focuses on patients with comorbidity of SMI such as schizophrenia and psychosis, but none of the team members has such expertise. PI Brooks Carthon is trained as a psychiatric nurse, but her research does not address patients with SMI or comorbidity of SMI and non-mental health conditions. Several team members' expertise are overlapping. No data is provided about Ns and % of patients with SMI in the study site. The PI recently completed a similar pilot with the same intervention and the same population as proposed in the current proposal. The results of the pilot were insignificant. It is unclear how Aim 2 in the proposed application differs from what has been achieved. The justification for proposing the implementation of an insignificant intervention in the same population needs to be clarified. It is also unclear why patients are not part of the workgroup in Aim 1.

1. Significance:
Strengths

- Individuals with SMI are likelier to have nonpsychiatric hospitalizations than the general population and experience worse outcomes. Individuals with SMI present to the hospital with poorly managed medical conditions compared with other patients and require more intensive transitional care coordination after a hospitalization.
- Over a third of THRIVE participants hospitalized for a medical condition had a co-occurring SMI diagnosis.
- Interviews with five THRIVE participants with SMI revealed that they avoided engagement around mental health needs due to concerns over stigma and limited connections to behavioral health resources.

Weaknesses

- While the team argues that readmission rates for adult Medicaid patients age 45-64 are higher, the team presents data from 2014 indicating only a 4% difference compared to older adults and those with disabilities insured by Medicare (reference #21). It is not clear if

this difference is significant, and regardless, updated literature is needed to support the argument.

- The population of focus is unclear; is it all people who are insured by Medicaid? Or those who are insured by Medicaid and have SMI? Later in the proposal, there is a focus on Black patients, but in the Significance, it is unclear if the focus is on Black individuals or all Medicaid beneficiaries.
- The team presents THRIVE as a means to address unmet needs, but no description of these unmet needs is provided.
- A pilot study comparing THRIVE participants with (n=43) and without SMI (n=75) in readmission and ED outcomes did not reach statistical Significance.
- No clear description of THRIVE is offered. For example, only in the Innovation, it is mentioned that THRIVE leverages technology with an EHR “flag,” but there is no previous description of this intervention element.
- These weaknesses reduce enthusiasm that the project will likely result in D&I evidence that can contribute to a better understanding of how to scale the intervention or apply it to other settings and populations.

2. Investigator(s):
Strengths

- PI, Margo Brooks Carthon, PhD, APRN, an Associate Professor of Nursing and Africana Studies at the University of Pennsylvania, has suitable competency with respect to disparities and equity research; She is an adult psychiatric nurse by training, her research addresses health disparities among Black adults, and she is one of the developers of the THRIVE Clinical Pathway, the intervention studied in this proposal. PI also devotes 3 CM in each given year of the project.

Weaknesses

- While PI Brooks Carthon worked as a psychiatric nurse, her research does not address SMI and the team lacks expertise in this content area, which is critical to the proposal.
- Less clarity and justification are provided about the contribution of the other core team members. The team includes one or two PIs (unclear, see below) and 6 or 7 Co-Is. Several of the Co-Is' expertise overlap: Co-I Brom supports implementation, and so does Co-I Bettencourt. Co-I Cacchione served on the original interdisciplinary workgroup that developed THRIVE, like other team members, but her specific contribution to this project is unclear. Co-I Lasater's role overlaps with Co-I Harhay's focus on quantitative analysis of the trial. The work of Co-I Leary focuses on cardiac arrest using innovative technologies and on Human-centered Design, and as the Director of Innovation at the University of Pennsylvania's School of Nursing, it is unclear how her experience is relevant to the proposed project and why she is qualified to serve as key personnel.
- The role of PI (or Co-I?) Brom is unclear; while on the first page of the application, it is defined as a subaward PI, in her Biosktech she identified as a co-investigator. It is also unclear how Brom will contribute to the adoption, implementation, and evaluation of THRIVE at a new site, while she has no affiliation with the Hospital of the University of Pennsylvania –Cedar Avenue (HUP-Cedar).
- It is unclear why some Co-Is who contribute expertise for a specific Aim are budgeted throughout the entire 3-year project (e.g., Bettencourt, Cacchione, Lasater)

- The contribution of Dr. Nikpour as a co-investigator is unjustified and overlaps with the expertise of Co-I Lasater and Co-I Harhay. The description of the activities Of Co-I Nikpour also overlaps with the responsibilities of Mr. Jesse Chittams (Data Analyst) and Mr. Irene Hung (Research Coordinator).

3. Innovation:
Strengths

- More research on behavioral health interventions for patients with SMI in primary care is needed

Weaknesses

- The proposed approaches to address contextual factors impacting inequities are not innovative.
- A participatory approach is needed but not innovative.
- Virtual case management conferences are not innovative in mental health.
- People diagnosed with SMI are often excluded from research participation, but at the same time, there are many research projects of co-production and collaboration with individuals with SMI.
- The PI recently completed a similar pilot.

4. Approach:
Strengths

- The study will employ a Type I Hybrid effectiveness-implementation stepped wedge cluster randomized controlled trial
- In Aim 3, the team will attempt to recruit 40 patients for interviews (20 with SMI and 20 without).

Weaknesses

- The PI was also a PI on a recently completed 2-year project (ONR Penn Nursing University of Pennsylvania) that explored the outcomes of Medicaid-insured individuals diagnosed with SMI compared to those without SMI diagnoses and receiving services through the THRIVE clinical pathway. It is unclear how Aim 2 in the proposed application differs from what has been achieved in the ONR project.
- A contribution of the Community Advisory Board to the adaption and development of the study is warranted and required by this NOFO, but the description in the Approach (including the LoS) doesn't describe the plan to center equity and ensure the voices of stakeholders and community members with co-occurring SMI, and those from HUP-Cedar. There are concerns if the expectation for “meaningful engagement, feedback, and 1:1 participation on a quarterly basis and for protocol and toolkit development, and participation in dissemination” could be met with a compensation of $200 annually.
- The suitability of the study site (HUP-Cedar) is questioned as the team did not provide data on the Medicaid, racial, ethnic, and socioeconomic distribution and number of its current patients. No information is provided on the number of patients with co-occurring SMI. The team describes that the location of the hospital is in an area with predominantly Black residents who are publicly insured, but this doesn't help determine what the actual population in HUP-Cedar is, and if the study site is relevant for this NOFO and if the sample size in Aim 2 can be achieved.

- Since THRIVE was not found effective in a pilot with a decent number of participants (n=181), the rationale and motivation for a Type I Hybrid effectiveness-implementation is unclear. An RCT effectiveness design would be better suited, as the intervention had not been established as effective, yet.
- It is unclear why in Aim 1, a participatory aim, no patients with co-occurring SMI are part of the workgroup.
- It is unclear why the constant comparative analysis was chosen, as the purpose of this method is to compare new data with previously collected data, looking for similarities and differences. This Approach does not fit the goal in aim 1, which is to identify components of the EOHC framework that could be integrated into THRIVE and then evaluate the context required for adaptation of the THRIVE. Thematic analysis methods would be more appropriate, focusing on examining themes or patterns of meaning within data.
- In Aim 2, it is unclear why SMI is not an inclusion criterion. It is also unclear what is the potential pool of co-occurring SMI in the study site and how the team will ensure enough participants with co-occurring SMI.
- The equity-relevant metrics included in implementation and outcome evaluation are based on medical chart data. It is unclear if the metrics include measures of health equity and both direct and indirect effects of contextual factors impacting health equity.
- In the section of primary analysis for Aim 2, the team estimates that a 1/3 of the patients will have SMI, but since the focus is on adapting THRIVE to those with SMI, there is no description of how randomization will be conducted to ensure a focus on those with SMI and THRIVE vs. those with SMI and no THRIVE.
- In the Timeline Table, it is unclear how the team can develop the THRIVE adaption in Year 1 Q1, while the analysis of the interviews will be conducted in Year 1 Q2.

5. Environment:

Strengths

- The study will be conducted at the Hospital of the University of Pennsylvania –Cedar Avenue (HUP-Cedar), a small hospital with 100 beds that was recently acquired by the UPHS health system in 2021. The hospital is located in west Philadelphia, a geographical area with approximately 75% of whom are Black.

Weaknesses

- HUP-Cedar is owned and operated by The Public Health Management Corporation, not Penn Medicine, which only provides remote medical services. None of the 8 team members is affiliated with Public Health Management Corporation, and none is affiliated with HUP-Cedar. Therefore, concerns exist regarding the feasibility of this study, particularly as this is an implementation study that requires constant collaboration with the implementation site (HUP-Cedar) and engages staff in research activities.
- No description is provided about the racial/ethnic distribution and the socioeconomic status of HUP-Cedar. Therefore, although its location is in a geographical area with approximately 76% of whom are Black individuals, and over 80% are publicly insured, there is no information on how many of HUP-Cedar's patients are socioeconomically disadvantaged Black patients.
- The site, HUP-Cedar, is a small hospital with 100 beds. It is unclear if it has a psychiatric unit. Because the focus is on patients with SMI, these patients are usually seen in psychiatry, not primary care. No information is provided about the psychiatric unit in HUP-

Cedar. In such a small 100 beds hospital, the psychiatric unit should be very small (if it even exists), and it is unclear how the team can expect to enroll 472-534 patients with SMI in 8 months.

Protections for Human Subjects:
Acceptable

Data and Safety Monitoring Plan:
Acceptable

Single IRB for Cooperative Research:
Acceptable

Inclusion of Priority Populations:
Acceptable

Inclusion of Women:
Acceptable

Inclusion of Minorities:
Acceptable

Degree of Responsiveness:
The application is partly responsive

Budget and Period of Support:
Acceptable

Resource Sharing Plans:
The Data Management Plan is reasonable

CRITIQUE 2

Significance:	4
Investigator(s):	1
Innovation:	6
Approach:	7
Environment:	1

Overall Impact: This proposal emphasizes the importance of care transitions for patients with serious mental health concerns. Dr. Brooks Carthon is an exceptionally prepared PI, and the assembled investigative team is individually experienced and has effectively collaborated previously. This study tackles an important weakness in the US healthcare system, and seeks to evaluate efforts with a rigorous multi-level approach. Weaknesses in the design and limited innovation beyond existing programming diminishes enthusiasm.

1. Significance:
Strengths

- Transitions from a care environment to home can be complex and overwhelming, particularly for patients dealing with SMI. The proposed work seeks to improve these transitions and enhance continuity of care
- Previous evaluation of THRIVE program showed considerable efficacy

Weaknesses

- Difficult to determine what is actually new in this proposal beyond the evaluation plan
- The discussion of incorporating equity into THRIVE is somewhat nebulous
- The assertion, on the aims page, that a higher readmission rate for patients on Medicaid with SMI than comparators imply they are getting sicker rather than improving is confounded in a variety of ways.
- The conceptual framework serving as a backbone to the proposal seems unclear.

2. Investigator(s):
Strengths

- This is an exceptional PI and investigative team capable of successfully completing the proposed project
- Broad, complementary skill sets observed across the investigative team
- Considerable support for researchers at UPenn is evident

Weaknesses

- No significant concerns identified

3. Innovation:
Strengths

- The focus on SMI in care transitions represents a step forward in the literature

Weaknesses

- Majority of the proposal seems to be focused solely on evaluation, rather than substantial adaptation/expansion/refinement
- Creation of an EHR flag is not innovative/novel
- Much of the study seems like a replication/minor extension of Brooks Carthon et al (2022) in BMJ Open Quality. More distinction is needed

4. Approach:
Strengths

- Provision of a planned cost-benefit analysis of THRIVE efforts is a major strength, showing rigor and practicality
- Strong preliminary data implying efforts to decrease readmissions and related healthcare outcomes will be successful.

Weaknesses

- The study is powered on comparisons between THRIVE vs. no THRIVE and SMI vs. no SMI. This does not entirely represent the purported focus of the study, as the interaction between experimental condition and serious mental illness seems to be the most important/salient question at hand. Showing that individuals with SMI perform significantly better on outcomes in the THRIVE condition over the no THRIVE condition would better align with the presented equity focus of the study.
- The comparison groups are not equivalent as there seems to be some inherent self-selection issues. Patients in the THRIVE condition have to consent to participate while controls are simply selected from medical records.
- The limitations associated with relying solely on EHR review for utilization is mentioned very briefly, but no solutions or mitigation efforts are mentioned.
- Asking for extended supervision of patients from discharging physicians is asking acute care providers (like ED providers, urgent care doctors) to do even more than currently needed because other providers in the system have struggled. This could be a tough sell should broad implementation be planned.
- Relatedly, generalizability of findings seem fairly limited to study environment.

5. Environment:
Strengths

- Outstanding research institutions involved
- Substantial departmental/school support for nursing-related research
- Investigators hold important leadership positions at the proposed sites, enhancing the likelihood that additional support will be provided, if needed
- Access to the Biostatistics Evaluation Collaboration Consultation Analysis Lab enhances the already strong biostatistical skills among the investigators.

Weaknesses

- No concerns

Protections for Human Subjects:
Acceptable

Data and Safety Monitoring Plan:
Acceptable

Single IRB for Cooperative Research:
Acceptable

Inclusion of Priority Populations:
Acceptable

Focus on providing equitable services to patients with serious mental illness

Inclusion of Women:
Acceptable

Inclusion of Minorities:

Acceptable

Degree of Responsiveness:
Moderately responsive

Budget and Period of Support:
Acceptable

Resource Sharing Plans:
Reasonable

CRITIQUE 3

Significance:	5
Investigator(s):	2
Innovation:	1
Approach:	5
Environment:	1

Overall Impact: The application by Brooks Carthon and their expert team seeks to adapt an evidence-based transitional care pathway for Medicaid-insured individuals with multiple chronic conditions, THRIVE, to better serve patients with co-occurring serious mental illness (SMI) and evaluate it in a new hospital setting. The research aims were to (1) adapt THRIVE to be more equity- and SMI-focused through participatory approaches; (2) evaluate the adapted THRIVE vs. usual care on long-term healthcare utilization, stratified by SMI status; and (3) assess antecedent implementation outcomes, cost, and equity-focused determinants of implementation. This was a well-written application with many major strengths, including strong justification for this population, strong preliminary studies, and strong and innovative integration of various health equity frameworks that could serve as a model for future studies to follow. Despite also having strong implementation research methods, several minor and moderate weaknesses slightly dampen my enthusiasm. Of primary concern, given that the original THRIVE intervention was already effective for individuals with Medicaid insurance with SMI, this study felt like an incremental optimization, meaning the knowledge to be gained after a 3-year study is only moderately impactful/contribution. Second, given the stepped-wedge implementation occurs all within one hospital, contamination effects seem a likely threat to internal validity. More justification is also needed for the large investigative team over a shorter project pierod.

1. Significance:
Strengths

- The focus is on post-hospitalization disparities among Medicaid-insured individuals – economically disadvantaged individuals accessing care that is systemically deficient contributes to a cycle of health disparities and huge costs to the healthcare system.
- Well-described lack of interventions to address disparities in posthospitalization outcomes for Medicaid-insured individuals with medical and serious mental illness and deficiencies in current transitional care interventions.

- Builds directly on prior work comparing THRIVE participants with and without co-occurring SMI – the implementation gap.
- Original THRIVE (for people with multiple chronic conditions) demonstrated 50% decrease in 30-day readmissions and ED utilization and 50% increase in access to post-discharge care - evidence of effectiveness.

Weaknesses

- Given that the original THRIVE intervention worked for people with SMI (and seemingly slightly better than for people without SMI), another *effectiveness*-focused study seems too incremental and requires more rationale. It was unclear how different HUP-Cedar was from the original setting that the investigators felt more effectiveness data are needed. The proposed adaptations seem to be about optimization for people on Medicaid with SMI. From a research need perspective, a more implementation-focused study, even a later-stage hybrid study, could have greater impact on speeding up translation of this EBI intro widespread practice.

2. Investigator(s):
Strengths

- PI has strong funding background and mixed-methods expertise in linking nursing care and racial inequities; developer and lead of THRIVE intervention.
- Team has incredibly strong background in nursing research with diverse methods, including implementation science, cost evaluation, data management, statistical analysis, and equity-based design. Many investigators were part of the original development of THRIVE.

Weaknesses

- Despite a strong letter of support, no one on the investigative team is employed by the implementing health system and/or no one from HUP-Cedar is on the investigative team. Given the embedded nature of the work, this could affect feasibility of implementation.
- There is some overlapping expertise among the investigative team, and it is not 100% clear the role/contribution each person plays, particularly Drs. Brooks Carthon and Brom.

3. Innovation:
Strengths

- Prevailing transitional care interventions are focused on individual-level behavior change; THRIVE challenges this paradigm with a more multilevel approach, including system-level changes like EHR flags, virtual case management, and extended continuity of care.
- Incorporation of multiple equity frameworks together is particularly innovative.
- Focus on individuals with SMI, a population often excluded from research participation.

Weaknesses

- None noted by Reviewer.

4. Approach:
Strengths

- Well-thought-out incorporation of the Brownson health equity recommendations, health equity implementation framework, and equity-oriented health care framework.

- The collaborative workgroup has input throughout the process, especially in design of the adaptations.
- Strong epidemiological and intervention preliminary studies set up the current study.
- Given that there are only 4 nurse case managers, the stepped-wedge design seems appropriate to increase analytic power.
- Strong data collection and analysis plan.
- Cost analyses will be very important for future implementation.

Weaknesses

- Because case managers are in the same hospital, It was unclear how the investigators would prevent spillover/contamination effects, especially if case managers share clients /cover for each other, for patients that may encounter each other or for staff that may have to work across conditions.
- Three years seems a short time period for the three aims.
- The design is powered on THRIVE vs. usual care, but the focus of the study seems to be on THRIVE participants with SMI (so THRIVE among people with SMI vs. without SMI). It was unclear why the power calculation was not focused on the interaction effect of THRIVE x SMI instead.
- The lack of pharmacy/pharmaceutical use data seems like a potential missing confounder with regards to individuals with SMI, as it may differentially affect ED and hospitalization utilization for those individuals.
- Given that policy is a major outer setting determinant of Medicaid, engaging policy-level stakeholders seems particularly important for scalability, especially outside Pennsylvania settings. Despite the investigative team's expertise in this area, the only mention of policy's role seems to be in the data sharing plan (i.e., a potential policy brief as part of dissemination).

5. Environment:
Strengths

- Excellent academic resources at Penn (School of Nursing, BECCA Lab, Center for Health Outcomes and Policy Research, Leonard Davis Institute for Health Economics) and Villanova University as well as practice/community resources at Penn Health System (Perelman School of Medicine, Hospital @ Cedar Campus, Penn Medicine at Home, Penn Center for Community Health Workers)
- Clear details describing how investigators will collaborate across institutions

Weaknesses

- None.

Protections for Human Subjects:
Acceptable

Data and Safety Monitoring Plan:
Acceptable

Single IRB for Cooperative Research:
Unacceptable

The clinical trial in a new hospital system (study 1) is indicated to not be a multi-site study, so no single IRB attachment was included. However, investigators from multiple institutions are collaborating, suggesting cooperative research and thus the need for a single IRB. This does not align with how the application is framed so may be acceptable.

Inclusion of Priority Populations:
Acceptable

The primary priority population is low-income groups, specifically individuals on Medicaid.

Inclusion of Women:
Acceptable

Inclusion of Minorities:
Acceptable

Degree of Responsiveness:

This application is highly responsive to the NOFO.

Budget and Period of Support:

The budget is appropriate.

Resource Sharing Plans:

Data management and sharing plan is reasonable but very sparse in detail.

THE FOLLOWING SECTIONS WERE PREPARED BY THE SCIENTIFIC REVIEW OFFICER TO SUMMARIZE THE OUTCOME OF DISCUSSIONS OF THE REVIEW COMMITTEE, OR REVIEWERS' WRITTEN CRITIQUES, ON THE FOLLOWING ISSUES:

PROTECTION OF HUMAN SUBJECTS: ACCEPTABLE

INCLUSION OF WOMEN PLAN: ACCEPTABLE

INCLUSION OF MINORITIES PLAN: ACCEPTABLE

INCLUSION OF AHRQ PRIORITY POPULATIONS: ACCEPTABLE

INCLUSION ACROSS THE LIFESPAN: ACCEPTABLE

COMMITTEE BUDGET RECOMMENDATIONS: The budget was recommended as requested.

MEETING ROSTER

AHRQ Special Emphasis Panel
AGENCY FOR HEALTHCARE RESEARCH AND QUALITY
Dissemination and Implementation of Equity-Focused Evidence-Based Interventions in Healthcare Delivery Systems
ZHS1 HSR-B (02)
07/11/2023

CHAIRPERSON(S)

LINDER, JEFFREY, MD, MPH
PROFESSOR AND CHIEF
DIVISION OF GENERAL INTERNAL MEDICINE
FEINBERG SCHOOL OF MEDICINE
NORTHWESTERN UNIVERSITY
CHICAGO, IL 60611

MEMBERS

ABAR, BEAU, PHD
ASSOCIATE PROFESSOR
DEPARTMENTS OF EMERGENCY MEDICINE, PUBLIC HEALTH SCIENCES AND PSYCHIATRY
SCHOOL OF MEDICINE AND DENTISTRY
UNIVERSITY OF ROCHESTER MEDICAL CENTER
ROCHESTER, NY 14642

ASKANASE, ANCA, MPH, MD
PROFESSOR
DEPARTMENT OF RHEUMATOLOGY
COLUMBIA UNIVERSITY SCHOOL OF PHYSICIANS AND SURGEONS
NEW YORK, NY 10032

BETHEA, TRACI, MPA, PHD
ASSISTANT PROFESSOR
OFFICE OF MINORITY HEALTH AND HEALTH DISPARITIES RESEARCH
GEORGETOWN UNIVERSITY
GEORGETOWN LOMBARDI COMPREHENSIVE CANCER CENTER
WASHINGTON, DC 20003

BOWLES, NICOLE, MS, PHD
ASSISTANT PROFESSOR
OREGON INSTITUTE OF OCCUPATIONAL HEALTH SCIENCES
OREGON HEALTH AND SCIENCE UNIVERSITY
PORTLAND, OR 97239

ECHEVERRIA, SANDRA, PHD, MPH
ASSOCIATE PROFESSOR
DEPARTMENT OF PUBLIC HEALTH EDUCATION
SCHOOL OF HEALTH AND HUMAN SCIENCES
UNIVERSITY OF NORTH CAROLINA GREENSBORO
GREENSBORO, NC 27412

IVERS, LOUISE, MPH, MD
PROFESSOR AND CHAIR
GLOBAL HEALTH AND SOCIAL MEDICINE
HARVARD MEDICAL SCHOOL
HARVARD UNIVERSITY
BOSTON, MA 02115

KWAN, BETHANY, MPH, MA, PHD
ASSOCIATE PROFESSOR AND ASSOCIATE VICE CHAIR OF RESEARCH
DEPARTMENT OF EMERGENCY MEDICINE
UNIVERSITY OF COLORADO ANSCHUTZ MEDICAL CAMPUS
AURORA, CO 80045

LI, DENNIS, MPH, PHD
ASSISTANT PROFESSOR
DEPARTMENT OF PSYCHIATRY AND BEHAVIORAL SCIENCES
FEINBERG SCHOOL OF MEDICINE
NORTHWESTERN UNIVERSITY
CHICAGO, IL 60611

MORRATO, ELAINE, DRPH, MPH
FOUNDING DEAN AND PROFESSOR
PARKINSON SCHOOL OF HEALTH SCIENCES
AND PUBLIC HEALTH
LOYOLA UNIVERSITY CHICAGO
UNIVERSITY OF COLORADO ANSCHUTZ MEDICAL CAMPUS
CHICAGO, IL 60660

PASUPATHY, KALYAN, PHD, MS
PROFESSOR AND HEAD
DEPARTMENT OF BIOMEDICAL AND
HEALTH INFORMATION SCIENCES
UNIVERSITY OF ILLINOIS CHICAGO
CHICAGO, IL 60612

THORSEN, ANDREAS, PHD
ASSOCIATE PROFESSOR
DEPARTMENT OF BUSINESS MANAGEMENT
JAKE JABS COLLEGE OF BUSINESS & ENTREPRENEURSHIP
MONTANA STATE UNIVERSITY
BOZEMAN, MT 59717

VIGLIANTI, ELIZABETH, MPH, MD, MS
ASSISTANT PROFESSOR
DEPARTMENT OF PULMONARY AND CRITICAL CARE
UNIVERSITY OF MICHIGAN
ANN ARBOR, MI 48109

ZISMAN ILANI, YAARA, MA, PHD
ASSISTANT PROFESSOR
SOCIAL AND BEHAVIORAL SCIENCES
COLLEGE OF PUBLIC HEALTH
TEMPLE UNIVERSITY
PHILADELPHIA, PA 19122

SCIENTIFIC REVIEW OFFICER

BOGLE, XAVIER, PHD
SCIENTIFIC REVIEW OFFICER
DIVISION OF SCIENTIFIC REVIEW
OFFICE OF EXTRAMURAL RESEARCH, EDUCATION AND PRIORITY POPULATIONS
AGENCY FOR HEALTHCARE RESEARCH AND QUALITY
ROCKVILLE, MD 20857

JOHNSON, KEAIRA
PROGRAM ANALYST
DIVISION OF SCIENTIFIC REVIEW
OFFICE OF EXTRAMURAL RESEARCH, EDUCATION AND PRIORITY POPULATION
AGENCY FOR HEALTHCARE RESEARCH AND QUALITY
ROCKVILLE, MD 20857

Consultants are required to absent themselves from the room during the review of any application if their presence would constitute or appear to constitute a conflict of interest.
